# Matched comparison of decellularized homografts and bovine jugular vein conduits for pulmonary valve replacement in congenital heart disease

**DOI:** 10.1007/s10561-023-10082-4

**Published:** 2023-03-14

**Authors:** Dmitry Bobylev, Alexander Horke, Murat Avsar, Tomislav Cvitkovic, Dietmar Boethig, Mark Hazekamp, Bart Meyns, Filip Rega, Hitendu Dave, Martin Schmiady, Anatol Ciubotaru, Eduard Cheptanaru, Vladimiro Vida, Massimo Padalino, Victor Tsang, Ramadan Jashari, Günther Laufer, Martin Andreas, Alexandra Andreeva, Igor Tudorache, Serghei Cebotari, Axel Haverich, Samir Sarikouch

**Affiliations:** 1 Department for Cardiothoracic, Transplant, and Vascular Surgery, Hannover Medical School, Hannover, Germany; 2 Department of Congenital Cardiac Surgery, Leiden University Medical Center, Leiden, The Netherlands; 3 Department of Cardiac Surgery, Katholieke Universiteit Leuven, Leuven, Belgium; 4 Division of Congenital Cardiovascular Surgery, University Children’s Hospital, Zurich, Switzerland; 5 Cardiac Surgery Center, State Medical and Pharmaceutical University, Chisinau, Moldova; 6 Pediatric and Congenital Cardiac Surgery Unit, Azienda Ospedaliera di Padova, University of Padua Medical School, Padua, Italy; 7 Department of Cardiothoracic Surgery, Great Ormond Street Hospital for Children, NHS Foundation Trust, London, UK; 8 European Homograft Bank, Clinique Saint-Jean, Brussels, Belgium; 9 Department of Cardiac Surgery, Medical University of Vienna, Vienna, Austria

**Keywords:** Heart valve disease, Tissue engineering, Decellularization, Allografts

## Abstract

For decades, bovine jugular vein conduits (BJV) and classic cryopreserved homografts have been the two most widely used options for pulmonary valve replacement (PVR) in congenital heart disease. More recently, decellularized pulmonary homografts (DPH) have provided an alternative avenue for PVR. Matched comparison of patients who received DPH for PVR with patients who received bovine jugular vein conduits (BJV) considering patient age group, type of heart defect, and previous procedures. 319 DPH patients were matched to 319 BJV patients; the mean age of BJV patients was 15.3 (SD 9.5) years versus 19.1 (12.4) years in DPH patients (*p* = 0.001). The mean conduit diameter was 24.5 (3.5) mm for DPH and 20.3 (2.5) mm for BJV (*p* < 0.001). There was no difference in survival rates between the two groups after 10 years (97.0 vs. 98.1%, *p* = 0.45). The rate of freedom from endocarditis was significantly lower for BJV patients (87.1 vs. 96.5%, *p* = 0.006). Freedom from explantation was significantly lower for BJV at 10 years (81.7 vs. 95.5%, *p* = 0.001) as well as freedom from any significant degeneration at 10 years (39.6 vs. 65.4%, *p* < 0.001). 140 Patients, matched for age, heart defect type, prior procedures, and conduit sizes of 20–22 mm (± 2 mm), were compared separately; mean age BJV 8.7 (4.9) and DPH 9.5 (7.3) years (*p* = n.s.). DPH showed 20% higher freedom from explantation and degeneration in this subgroup (*p* = 0.232). Decellularized pulmonary homografts exhibit superior 10-year results to bovine jugular vein conduits in PVR.

## Introduction

In 2002, a glutaraldehyde cross-linked, heterologous bovine jugular vein conduit with a competent tri-leaflet venous valve was granted humanitarian use device (HUD) designation by the U.S. Food and Drug Administration. (Contegra Pulmonary Valved Conduit FDA Executive Summary 2020) Non-randomized multi-centre trials performed in the U.S. and Europe showed good results for this product, Contegra®, and identified several issues, such as distal graft stenosis, which were subsequently addressed by increased rinsing and modified suturing. (Boethig et al. 2012; Breymann et al. 2009) Numerous publications have described good results for bovine jugular vein (BJV) conduits when used for pulmonary valve replacement (PVR). Interestingly, Contegra is one of the very few products in cardiovascular medicine which is licensed only for paediatric use. It is nevertheless also used extensively for adults and has shown good results for this indication. (Boethig et al. 2009) There, however, is a size limitation for BJV in adults as the device is available in 6 sizes in even increments between 12 and 22 mm inside diameter, measured at the inflow end.

BJV and classic cryopreserved homografts (CH) have been the two most widely used options for pulmonary valve replacement (PVR) in congenital heart disease over the last two decades. Comparisons of Contegra® with CH have been reported on in a multitude of publications and BJV have shown similar, but slightly inferior results in most studies. (Marathe et al. 2021; Patel et al. 2020; Sandica et al. 2016; Yong et al. 2015) As a consequence, cryopreserved homografts have continued to be viewed by the majority of cardiac surgeons as the gold standard.

In 2013, fresh non-cryopreserved decellularized homografts were approved for use in pulmonary valve replacement by the Paul-Ehrlich-Institute, the German authority for human tissue-based devices. Five-year data from European-wide ESPOIR-trial, which started in 2014, showed excellent performance for decellularized pulmonary homografts (DPH) and low rates of adverse events. ESPOIR Registry data, covering a period of up to 15 years and including a matched comparison with CH, demonstrated statistically significant superior freedom from explantation and less dysfunction. (Bobylev et al. 2022).

The long-term durability of any biological conduit for pulmonary valve replacement depends on the age of the recipient, as immune response is increased in children, and also the type of congenital heart defect. (Khanna et al. 2015) PVR in an anatomical position, such as in the Ross procedure, has shown superior durability compared with extra-anatomical conduits. (Meijer et al. 2019) It also has been shown that a suboptimal outcome in the immediate postoperative phase, will result in lower freedom from re-intervention, even when the residual gradient or regurgitation is mild. (Bokma et al. 2015) Suboptimal surgical results are far more likely in re-do procedures.

The aim of this study is a matched comparison of bovine jugular vein conduits and decellularized homografts considering patient age, type of congenital heart defect, and the number of previous heart operations.

## Materials and methods

### Cohorts for matching

Patients who had received a decellularized pulmonary homograft (DPH) were recruited from the ESPOIR Registry. The registry aims to provide follow-up on all patients receiving DPH processed by corlife oHG (www.corlife.eu), a Hannover-based biotechnology company which provides decellularization as a service to tissue establishments. At the time of this analysis, the registry contained data on 361 DPH patients, amounting to approximately 90% of all processed DPH which have been implanted to date, including 121 patients from the prospective ESPOIR trial. Recently, the 5-year results of this trial were published including a comparison of decellularized fresh homografts with conventional cryopreserved homografts based on the above-mentioned registry data. (Bobylev et al. 2022).

BJV patients for matching were chosen from the updated RVOT Conduit Registry (Sandica et al. 2016). Matching was performed on the basis of the patient’s age category at implantation, the type of congenital heart defect, the number of previous operations, and the number of previous PVR. In 42 out of the 361 total patients with DPH, no match was found within the RVOT Conduit Registry, which reduced the number of DPH patients to be analysed to 319 for whom matches were available.

56% of all patient data sets analysed were derived from the seven centres participating in the ESPOIR trial; the remaining were derived from seven additional centres participating in the RVOT Registry. BJV patients were younger to a statistically significant degree due to the upper size limit of 22 mm of BJV. Therefore, an additional comparison was performed in patients who were additionally matched for conduit size. For this comparison, only matched patients with conduit sizes of 20–22 mm (± 2 mm) were included.

A peak echocardiographic gradient of ≥ 50 mmHg and regurgitation grade ≥ moderate was defined as dysfunctional.

The ESPOIR trial was registered under ClinicalTrials.gov, NCT 02,035,540, and results have been published at the respective ClinicalTrials.gov website. Approval was given by all local ethics committees (22.10.2013, No. 2016/2013) before the start of the study, and informed consent was obtained appropriately from all participants or parents.

### Statistics

Summaries of the numeric data are given as means and standard deviation. The Mann–Whitney U test for non-paired samples was used for data with skewed distribution. Time-related events, such as freedom from explantation and endocarditis, were evaluated according to Kaplan–Meier, including numbers at risk at 0, 5 and 10 years, as well as freedom-from-event rates at 0, 5 and 10 years, with their respective 95% confidence limits.

The proportion of explanted and dysfunctional grafts over time was calculated for the two valve grafts as described above, with linear interpolation of the numerical values for peak gradients and insufficiency grades. The yearly status fractions were multiplied with the explantation rates observed at the end of the entire post-implantation rates. ^3^ The Kruskall-Wallis-test for independent samples with sorted hypotheses was applied to compare the fractions of intact conduits.

SPSS 23 (IBM Corporation, Somer, NY) was used for the analyses. No correction for multiple testing was performed. All the statistical tests were two-sided and a probability p-value of 0.05 or less was considered statistically significant.

## Results

### Outcome comparison of DPH recipients with matched bovine jugular vein conduit patients

319 DPH patients were matched to 319 BJV patients: the mean age of BJV patients was 15.3 (SD 9.5) years versus 19.1 (SD 12.4) years in DPH patients (*p* = 0.001). The mean conduit diameter of DPH was 24.5 (SD 3.5) mm and 20.3 (SD 2.5) mm for BJV (*p* < 0.001).

Table 1 provides details for the two study cohorts.

**Table 1:** Patient characteristics for the decellularized pulmonary homografts (DPH) cohort and the respective bovine jugular vein (BJV) cohort.

Implantation period	DPH	BJV
	2005–2020	1999–2016
*Diagnoses in %*		
TOF	45	60
Ross	13	8
PI/PS	14	8
PA	10	8
DORV	5	6
TAC	4	6
TGA	3	4
Other	6	0
Total (n)	319	319
Mean age at implantation [years, SD]	19.1 (12.4)	15.3 (9.5)
Mean follow-up [years, SD]	4.4 (3.2)	6.4 (4.3)
Total follow-up [years]	1408	1891
Sex (male)	200 (63%)	169 (53%)
*Number of previous operations:*		
0	49	25
1	153	182
2	82	75
> 2	35	37
Mean conduit diameter [mm, SD]	24.5 (3.5)	20.3 (2.5)
12–19 [mm]	26	79
20–23 [mm]	94	238
24–29 [mm]	199	2

*SD* standard deviation

There was no difference in survival rates for the two groups after 10 years (97.0 vs. 98.1%,*p* = 0.45). The rate of freedom from endocarditis was significantly lower for BJV patients (87.1 vs. 96.5%, *p* = 0.006). Freedom from explantation was also significantly lower for BJV at 10 years (81.7 vs. 95.5%, *p* = 0.001) as was freedom from any significant degeneration at 10 years (39.6 vs. 65.4%, *p* < 0.001).

Table 2 summarizes the specific results for each conduit type at 5 and 10 years.

**Table 2:**
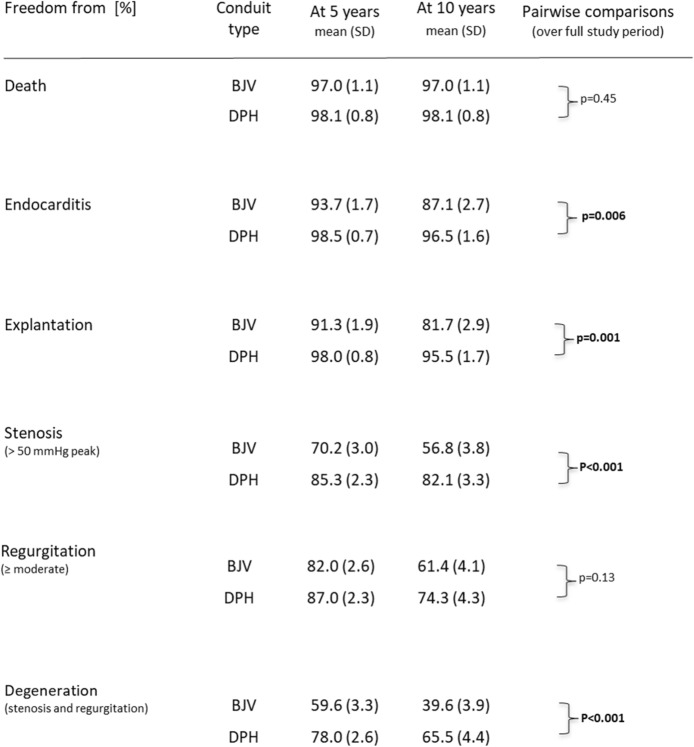
Freedom from diverse adverse outcomes in matched cohorts of 2 × 319 patients for decellularized pulmonary homografts (DPH) and bovine jugular vein (BJV) cohorts. SD: standard deviation

**Fig. 1:**
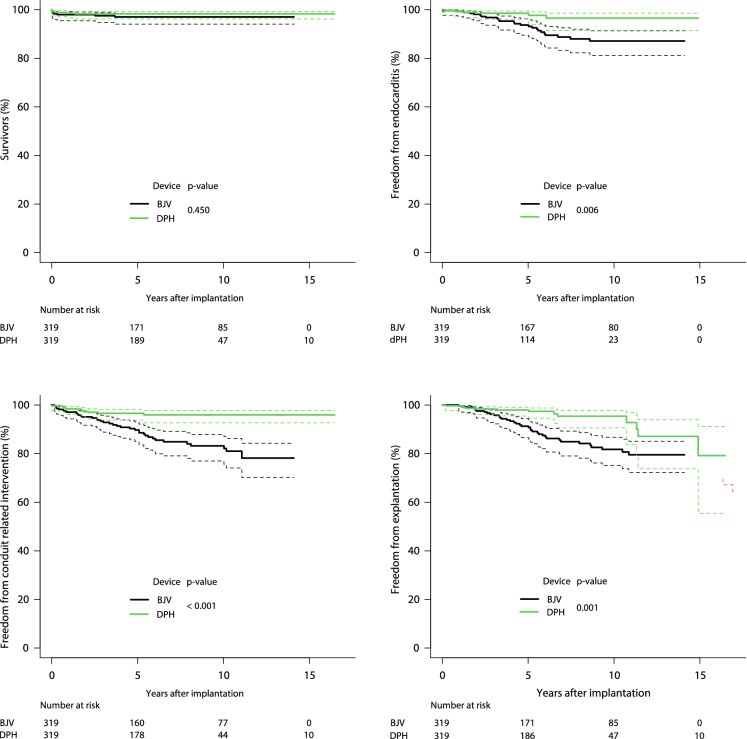
Kaplan–Meier curves for freedom from death, endocarditis, conduit-related catheter interventions and explantation for matched cohorts of decellularized pulmonary homografts (DPH) and bovine jugular vein conduits (BJV). *p*-values for pairwise comparisons are also given

**Fig. 2:**
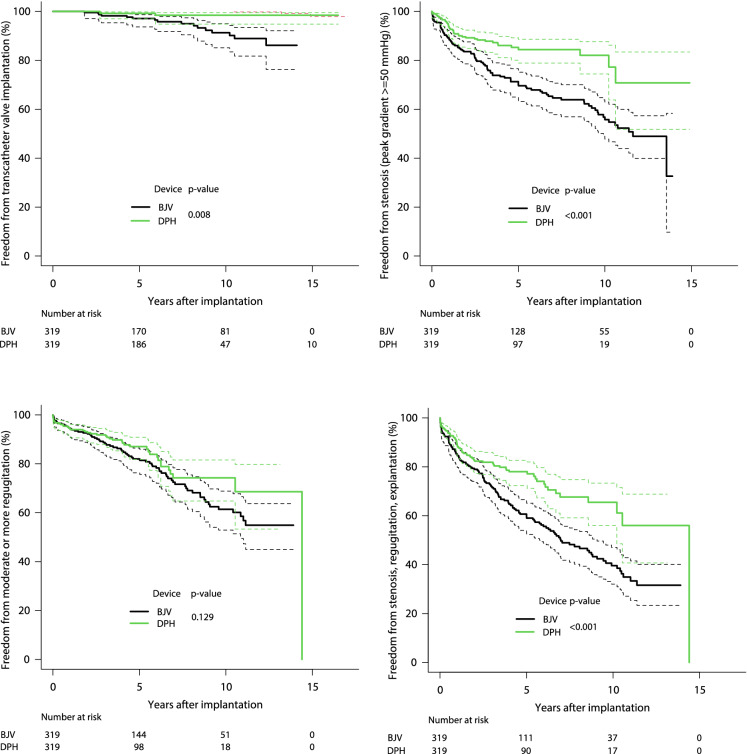
Kaplan–Meier curves for freedom from catheter valve implantation, stenosis, regurgitation, as well as freedom from combined degeneration and explantation. *p*-values for pairwise comparisons are also given

Figure 1 and 2 provide Kaplan–Meier analyses for freedom from death, endocarditis, conduit-related catheter interventions, explantation, catheter valve implantation, stenosis, regurgitation, as well as freedom from combined degeneration and explantation.

**Fig. 3:**
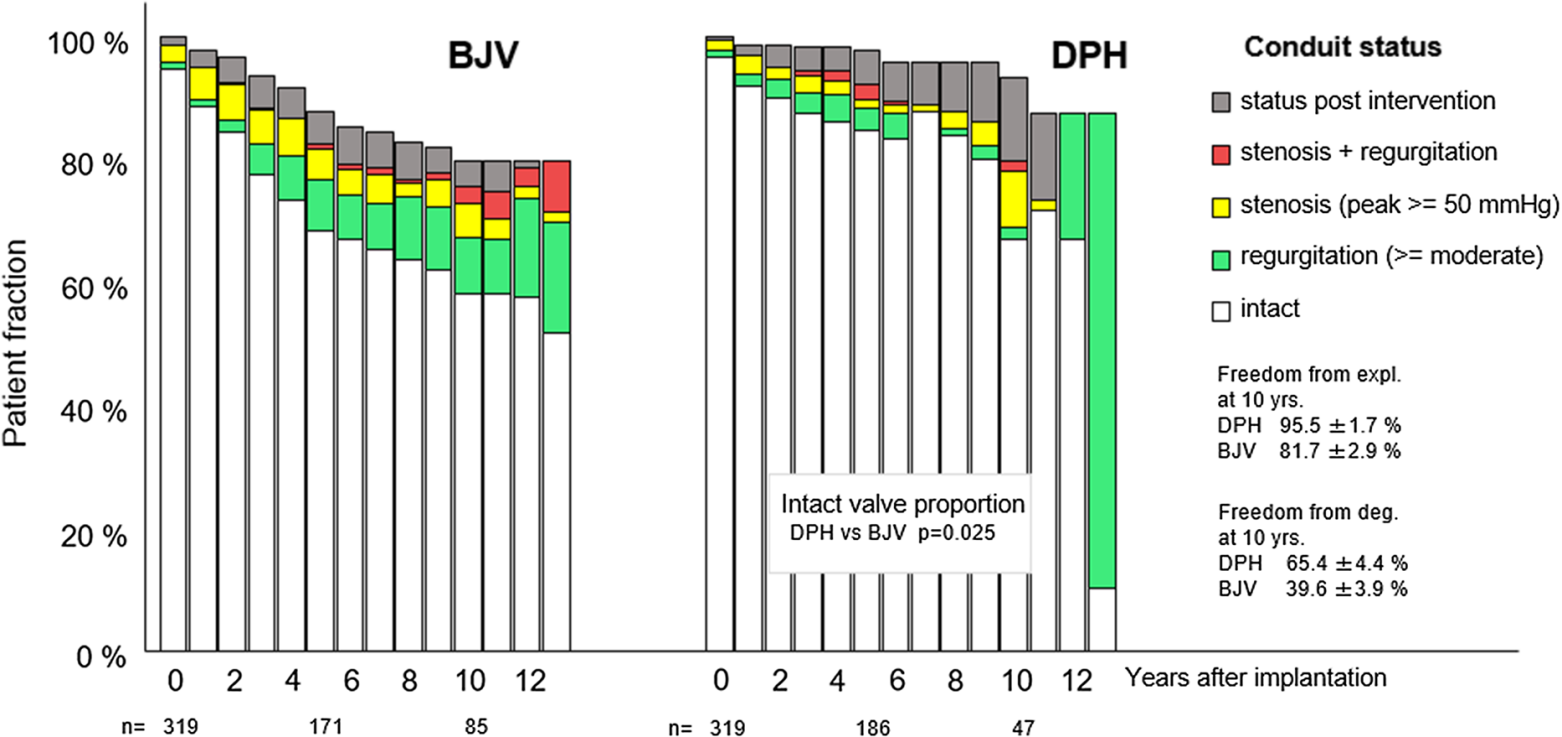
Freedom from explantation and functional conduit status for matched decellularized pulmonary homograft (DPH) and bovine jugular vein (BJV) cohorts. We also analysed whether the yearly summarized fractions of intact DPHs during all the commonly observed years were larger than these fractions of BJVs. Detailed pairwise comparisons including *p*-values are provided in Table 2

Figure 3 gives an overview of freedom from explantation and the current functional status of the remaining conduits within the two matched groups. Yearly summarized fractions of intact DPHs during all the commonly observed years were statistically significantly larger than the corresponding fractions of BJV (*p* = 0.025).

### Outcome comparison of DPH and BJV according to conduit size

**Fig. 4:**
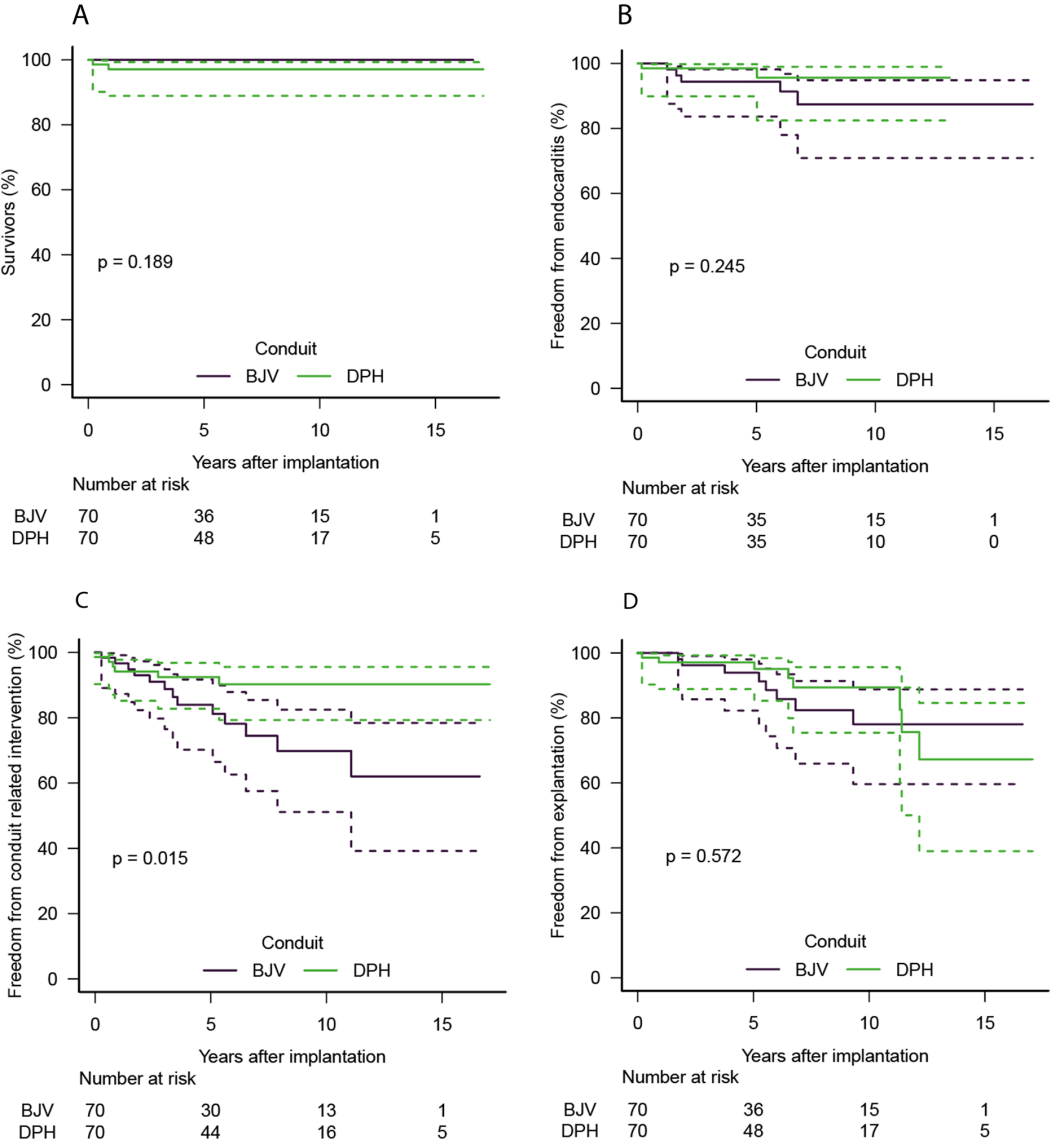
Kaplan–Meier curves for freedom from death, endocarditis, conduit-related catheter interventions and explantation for matched sub-cohorts of decellularized pulmonary homografts (DPH) and bovine jugular vein conduits (BJV), which were additionally limited to a comparison of grafts with 20–22 mm annulus diameter (± 2 mm). *p*-values for pairwise comparisons are also given

**Fig. 5:**
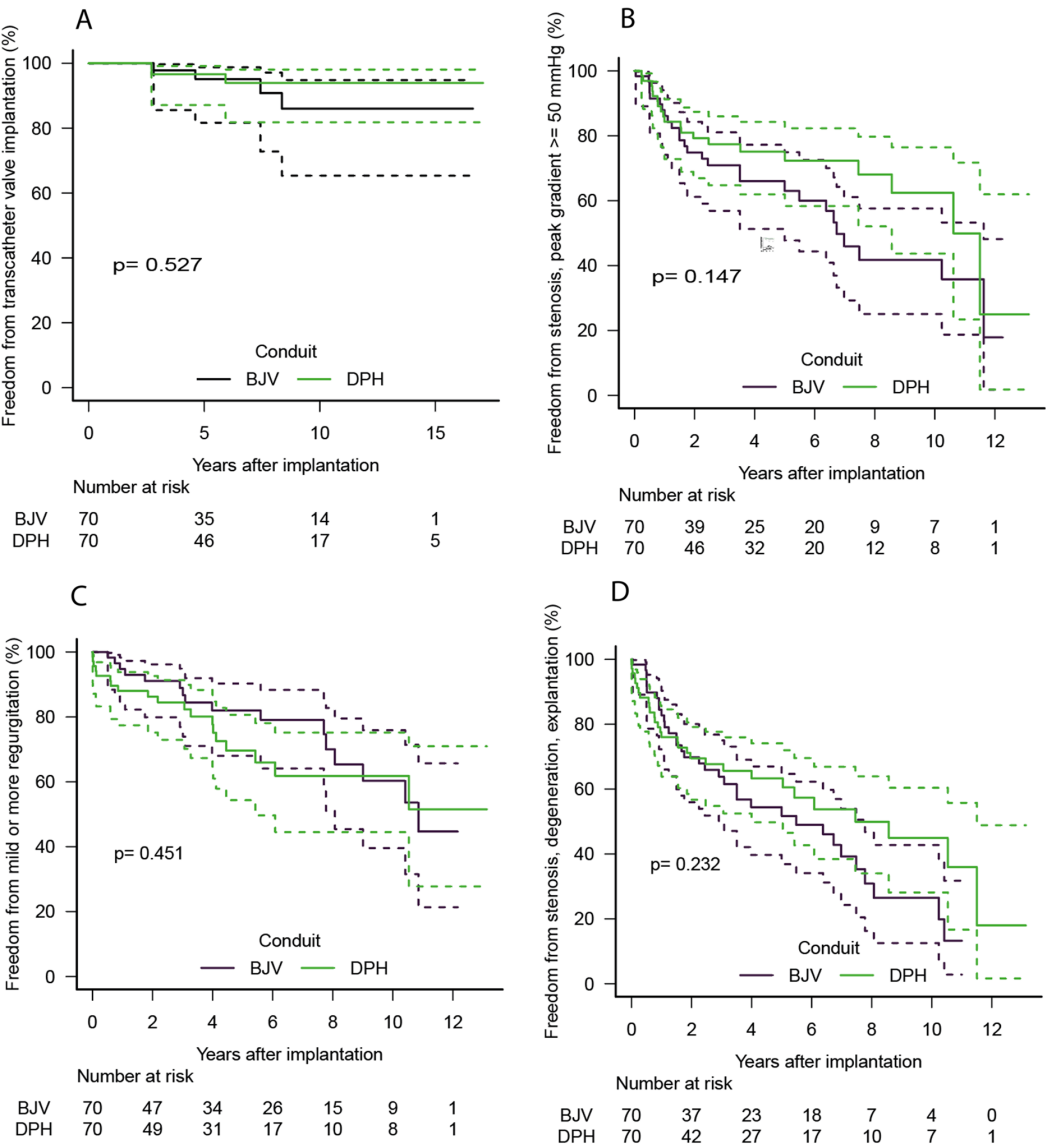
Kaplan–Meier curves for freedom from catheter valve implantation, stenosis, regurgitation, as well as freedom from combined degeneration and explantation. For matched sub-cohorts of decellularized pulmonary homografts (DPH) and bovine jugular vein conduits (BJV), which were additionally limited to a comparison of grafts with 20–22 mm annulus diameter (± 2 mm). *p*-values for pairwise comparisons are also given

140 Patients matched for age, heart defect, prior procedures and conduit sizes of 20–22 mm (± 2 mm) were compared in a separate analysis. Figures 4 and 5 provide Kaplan–Meier analyses for freedom from death, endocarditis, conduit-related catheter interventions, explantation, catheter valve implantation, stenosis, regurgitation, as well as freedom from combined degeneration and explantation. DPH showed 20% higher freedom from explantation and degeneration for DPH in this subgroup (*p* = 0.232). Table 3 provides details for these two sub-cohorts.﻿

**Table 3:** Patient characteristics for the additionally size-matched decellularized pulmonary homografts (DPH) sub-cohort and the respective bovine jugular vein (BJV) sub-cohort

Implantation period	DPH	BJV
	2005–2021	1999–2016
*Diagnoses in %*		
TOF	44	58
Ross	10	9
PI/PS	16	7
PA	10	7
DORV	5	9
TAC	3	6
TGA	4	4
Other	8	0
Total (n)	70	70
Mean age at implantation [years, SD]	9.5 (7.3)	8.7 (4.9)
Mean follow-up [years, SD]	7.5 (4.4)	6.3 (4.3)
Total follow-up [years]	374	389
Sex (male)	40 (57%)	37 (53%)
* Number of previous operations:*		
0	9	9
1	32	44
2	20	14
> 2	9	3
Mean conduit diameter [mm, SD]	19.8 (2.7)	18.6 (3.1)
12–19 [mm]	24	33
20–23 [mm]	46	37
24–29 [mm]	0	0

*SD* standard deviation

## Discussion

Bovine jugular vein conduits have together with cryopreserved homografts traditionally been the two main options for PVR with comparable performance. From a surgical viewpoint the intraoperative handling of both grafts is comparable as outlined by a publication from Wauthy et al., which found no statistically significant difference in the operative data for anesthesia, surgery, cardiopulmonary bypass, and aortic clamping durations. (Poinot et al. 2018).

We have shown the results of decellularized pulmonary homografts to be superior to those obtained in classic cryopreserved homografts for this indication using the 5-year results of the prospective multi-center ESPOIR trial and long-term data from the ESPOIR Registry in a matched comparison. (Bobylev et al. 2022) A recent meta-analysis confirmed significantly lower re-operation rates for decellularized homografts than for conventional cryopreserved homografts. Importantly, this meta-analysis used data from studies with different decellularization protocols. (Waqanivavalagi et al. 2020).

Within the present analysis, we demonstrate that results for decellularized pulmonary homografts (DPH) also indicate better performance than the results of bovine jugular vein conduits (BJV). The most notable difference may be the significantly higher freedom from endocarditis and the higher freedom from reoperation in matched patient cohorts. While one can argue that the two cohorts were not perfectly matched as Contegra® patients were younger, we do show that DPH resulted in about 20% better freedom from explantation and valve degeneration in exactly the same conduit sizes. The lack of statistical significance can be explained by the relatively low patient numbers in this subgroup analysis.

Fortunately, no difference was found in survival rates, which could be attributed to the robust monitoring system in Europe following surgery for congenital heart defects and experience with timely re-interventions. Endocarditis, however, showed significant differences with about 10% more patients affected after 10 years following BJV implantation. This result is corroborated by two recent nationwide registry-based analyses in Denmark and Germany, which found the risk for endocarditis in Contegra® and Melody® to be about five-to-six times higher than following pulmonary valve replacement using homografts (hazard ratios 6.72 and 5.49, *p* < 0.001, (Stammnitz et al. 2022)). This is a dramatic finding and has yet to be included in guideline recommendations. In our view, patients with an increased risk for infective endocarditis should not receive BJV in surgical or catheter-based procedures in view of the considerable morbidity and mortality rates. (Abdelghani et al. 2018 ).

The main advantage of valved bovine jugular vein (BJV) conduits over valved allografts is their better availability, as these prostheses do not rely on the complex pathway of human tissue donation. This holds especially true for the smaller sized grafts needed for pulmonary valve replacement in children and BJV have shown similar results to crypopreserved homografts when used in newborns, infants, and small children.(Fiore et al. 2010) Some surgeons attribute this to the tendency of BJV to dilate, which allows for slightly longer freedom from reoperation. Unfortunately, the number of small DPH in this analysis was limited and did not allow for a robust statistical comparison to small-sized BJV. Hopefully, this comparison can be performed in the future as more tissue banks increasingly recognize the benefits of decellularization. (Jashari 2021).

Despite the better performance of DPH, these grafts can still elicit an immune response which may be stronger in younger patients. In decellularized aortic homografts there were markedly better results in adult recipients. (Horke et al. 2020) We have shown the high levels of variance in the binding of preformed antibodies to different homogenized decellularized homograft samples in healthy controls, which inspired the idea of pre-operative matching algorithms to improve results in younger patients. (Ebken et al. 2021) We are currently analyzing the humoral immune response towards decellularized homografts in patients pre-and postoperatively, with the aim of developing early screening methods for immunological activation.

At the same time, very little is known about the immunological mechanisms involved in BJV failure and there is sparse literature focusing on this aspect. Xenogenic tissue will elicit a strong immune response, which may be mitigated by glutaraldehyde cross-linking, but not eliminated. (Williams et al. 2021) Decellularized xenogenic tissues still elicit a strong immune response and this has led to dramatic clinical consequences. (Simon et al. 2003) Therefore, a possible option could be the combination of decellularization and cross-linking of xenogenic tissue. (Williams et al. 2021) Genetically modified animal sources may further allow for improved clinical performance of xenogenic tissues in general in the future. (Ramm et al. 2021).

Overall, bovine jugular vein conduits fare well when used for pulmonary valve replacement as indicated by a very low 10-year mortality. However, results for decellularized pulmonary homografts show better performance. Given the limited availability of human tissue, further research on xenogenic grafts seems inevitable but also promising in view of recent advances made in the genetic engineering of animals.

## Limitations

The restrictions inherent in the matching process as a result of low numbers and high variance in congenital heart defects are the main limitations of this analysis.

Only retrospectively collected data was available for BJV with, at times, incomplete data in echocardiographic reports. However, this would be more likely to lead to an underestimation of valve degeneration and other adverse events in the BJV cohort in comparison with the prospective follow-up for DPH patients.

## Conclusion

Decellularized pulmonary homografts exhibit better results than bovine jugular vein conduits in pulmonary valve replacement. Freedom from explantation in matched cohorts was higher for decellularized homografts due to less degeneration. Freedom from endocarditis was significantly reduced in bovine jugular vein conduits.

## Data Availability

The data underlying this article will be shared on reasonable request to the corresponding author.

## Abbreviations

BJV: Bovine jugular vein
CH: Cryopreserved homograft
DORV: Double outlet right ventricle
DPH: Decellularized pulmonary homograft
HUD: Humanitarian use device
PA: Pulmonary atresia
PI: Pulmonary insufficiency
PS: Pulmonary stenosis
PVR: Pulmonary valve replacement
RVOT: Right ventricular outflow tract
SD: Standard deviation
TAC: Truncus arteriosus communis
TGA: Transposition of great arteries
TOF: Tetralogy of Fallot
